# Poster 2022: Sublingual immunotherapy in the treatment of allergic rhinitis: series of cases

**DOI:** 10.1186/1939-4551-7-S1-P28

**Published:** 2014-02-03

**Authors:** Sandra Nora Gonzalez-Diaz, Alfredo Arias-Cruz, Lorena Rangel-Garza

**Affiliations:** 1 Universidad Autonoma de Nuevo Leon, Hospital Universitario, Monterrey, Mexico; 2 Allergy and Clinical Immunology, Universidad Autonoma De Nuevo Leon - Hospital Universitario “Dr. Jose Eleuterio Gonzalez”, Monterrey, Mexico; 3 Universidad Autonoma de Nuevo Leon, Hospital Universitario "Dr. Jose E. Gonzalez" UANL, Monterrey, Mexico

## Background

Sublingual immunotherapy (SLIT) has been regarded as a viable alternative to subcutaneous immunotherapy (SCIT) since its introduction in 1986. Several studies have demonstrated the efficacy of SLIT. The safety profile of SLIT is superior to that of SCIT. No fatalities have been reported, and severe systemic reactions are rare. The rate of adverse event (AE) after SLIT is variable in the reported studies, but local AEs are predominant. Systemic side effects such as rhinitis, asthma, urticaria, angioedema, and hypotension make up a minority of the adverse reactions because local reactions (oropharyngeal or gastrointestinal) are most frequently reported.

## Objectives

To determine the efficacy and safety of SLIT in allergic rhinitis patients treated in our clinic.

## Methods

This is a descriptive, observational an retrospective study that included all the records files of private practice patients during the last 2 years with allergic rhinitis sensitized to aeroallergens (mites, grass and weeds pollen) confirmed by positive skin prick test, to which SLIT was initiated by a daily scheme in maintenance doses and then all of the patients being evaluated monthly searching for adverse reactions.

## Results

A total of 20 patients were included, with ages from 4 to 35 years (average of 12.3 years), 11 (55%) females and 9 (45%) males, from which 17 (85%) were children’s and 3 (15%) adults, all with the diagnose of allergic rhinitis, the average time with SLIT was 21 months (+/- 9.4) finding important clinical improvement in all patients from the symptoms of allergic rhinitis. No adverse reaction was found, neither systemic or local at any time. All patients stick to their treatment with a complete scheme of SLIT.

## Conclusions

SLIT demonstrated to be a secure and efficient in this group of patients, both pediatric and adults with allergic rhinitis with nonadverse reactions and with significant clinical improvement.

